# A Flower Pollination Optimization Algorithm Based on Cosine Cross-Generation Differential Evolution

**DOI:** 10.3390/s23020606

**Published:** 2023-01-05

**Authors:** Yunjian Jia, Shankun Wang, Liang Liang, Yaxing Wei, Yanfei Wu

**Affiliations:** School of Electronics and Communication Engineering, Chongqing University, Chongqing 400044, China

**Keywords:** flower pollination algorithm (FPA), cross-generation, differential evolution (DE), external archive, roulette wheel, robot path planning

## Abstract

The flower pollination algorithm (FPA) is a novel heuristic optimization algorithm inspired by the pollination behavior of flowers in nature. However, the global and local search processes of the FPA are sensitive to the search direction and parameters. To solve this issue, an improved flower pollination algorithm based on cosine cross-generation differential evolution (FPA-CCDE) is proposed. The algorithm uses cross-generation differential evolution to guide the local search process, so that the optimal solution is achieved and sets cosine inertia weights to increase the search convergence speed. At the same time, the external archiving mechanism and the adaptive adjustment of parameters realize the dynamic update of scaling factor and crossover probability to enhance the population richness as well as reduce the number of local solutions. Then, it combines the cross-generation roulette wheel selection mechanism to reduce the probability of falling into the local optimal solution. In comparing to the FPA-CCDE with five state-of-the-art optimization algorithms in benchmark functions, we can observe the superiority of the FPA-CCDE in terms of stability and optimization features. Additionally, we further apply the FPA-CCDE to solve the robot path planning issue. The simulation results demonstrate that the proposed algorithm has low cost, high efficiency, and attack resistance in path planning, and it can be applied to a variety of intelligent scenarios.

## 1. Introduction

With the advancement and innovation of science and technology, including the Internet of Things and artificial intelligence, there are a large number of complex high-dimensional variable optimization problems in engineering and industrial applications that need resolution, including solving engineering optimization problems [1], energy-saving development and utilization [2,3], andpublic facility construction [4]. Continuous, linear, and simple problems are typically amenable to traditional optimization algorithms [5,6,7], and yet when exploring larger combinatorial optimization problems, there are often great limitations, namely, low efficiency, high cost, and high energy consumption, which are not conducive to the application of engineers, and the solution accuracy cannot reach the desired value [8]. As a result, numerous academics began to seek out alternative methods, and heuristic algorithms emerged. Heuristic optimization algorithms are developed on the basis of the characteristic of biological systems and physical theory, Darwinian evolution, the swarm behavior of insects or animals, such as ants [9], birds [10], and annealing in physical metallurgy [11]. Heuristic optimization algorithms have high search accuracy, efficiency, and strong robustness [12], which can be classified into three categories. The first category has strong balance ability between local search and global search, such as artificial bee colony (ABC) [13], social spider optimization (SSO) [14], and so on. The second category has superior global convergence and computation robustness, namely, the wolf pack algorithm (WPA) [15], dragonfly algorithm (DA) [16], while algorithm (WA) [17], etc. The third category has greater population density and searching efficiency such as particle swarm optimization (PSO) [18] and genetic algorithm (GA) [19]. Although the aforementioned traditional algorithms have good performance in some situations, they may lack the exploration ability to find better solution space [20,21,22,23].

In view of this, Yang et al. [24,25] proposed a flower pollination algorithm (FPA) on the basis of flowering plants, which has been widely applied in multi-objective optimization. To improve the search capability of flower pollination algorithms, numerous researchers have addressed the shortcomings of flower pollination algorithm search strategies of flower pollination algorithms. In 2016, Zhou et al. [26] proposed the elite opposition-based flower pollination algorithm (EOFPA), in which the elite opposition learning strategy utilized the optimal individual information to extend the search range of the algorithm, which helped to enhance the global superiority seeking ability of the algorithm and increased the probability of searching for excellent solutions. In 2018, Bian et al. [27] proposed the self-adaptive flower pollination algorithm (SFPA) to reduce the probability of falling into a local optimum, and the parameter control mechanism designed in the SFPA fluctuates within a certain range, thus adjusting the algorithm parameters. Nevertheless, the adjustment of the transition probability *P* is fluctuating on the basis of 0.8, which is a small fluctuation range and may not significantly improve the algorithm’s performance. In 2019, Supriya et al. [28] proposed an enhanced global-best-driven flower pollination algorithm (GFPA) to improve the convergence speed of the flower pollination algorithm. The GFPA introduces a search strategy with the best individual as the base individual, which gives the algorithm a chance to mine in the neighborhood of the best individual and increases the algorithm’s convergence speed. In 2020, Yang et al. [29] proposed an improved flower pollination algorithm with three strategies (IFPA) to enhance the convergence speed and optimality search accuracy of the algorithm. In the IFPA, the information of two adjacent bags of the majority of individuals is employed to guide the evolutionary direction, which is equivalent to providing two clear and promising directions for the algorithm search, and yet the similarity of two neighboring generations of optimal individuals results in a single overall trend of the algorithm search direction and an increased likelihood of falling into a local optimum. By analyzing relevant studies, the FPA can find the optimum of the objective function by global and local search, but some flaws remain unsolved: (1) The setting of parameters such as inertia weights affects the accuracy of the global search for the optimal solution. (2) The local search process lacks a search guide and falls into local optimum, resulting in lower search efficiency and convergence. (3) The complexity of the optimization problem increases the number of locally optimal solutions and hinders the FPA from obtaining optimal solutions.

To address these issue, we propose an improved flower pollination algorithm based on cosine cross-generation differential evolution (FPA-CCDE) to overcome the mentioned shortcomings of the FPA. The contributions of our work can be summarized as follows: (1) Setting the cosine inertia weight makes the global search initially strengthen the search ability at a faster rate and enhance the convergence speed of the algorithm. (2) The algorithm uses cross-generation differential evolution to guide individuals to approach the optimal solution, so that the local search process of the algorithm is guided. (3) The external archiving mechanism and the adaptive adjustment of parameters realize the dynamic update of the scaling factor and crossover probability to enhance the population richness as well as reduce the number of local solutions and then combine the cross-generation roulette wheel selection mechanism to reduce the probability of falling into the local optimal solution.

For the purpose of evaluating the superiority and robustness of the proposed FPA-CCDE, we compare it with the other five state-of-the-art heuristic optimization algorithms by benchmark functions in [30,31,32,33,34,35,36,37,38]. The experimental results indicate our algorithm has the best performance in terms of searching accuracy, convergence speed, and operation efficiency. Intending to evaluate the performance of the proposed algorithm in practical applications, we consider the robot inspection path planning issue as an illustration and design the inspection path in accordance with the optimization variables to verify the effectiveness of the algorithm. The experimental results prove the proposed algorithm can meet the requirements of low-cost, high efficiency and obstacle avoidance in inspection path planning.

The remainder of this paper is organized as follows. Section 2 introduces the main idea of the FPA. Section 3 describes the details of the proposed FPA-CCDE. Section 4 verifies the effectiveness of the FPA-CCDE by benchmark functions. Section 5 demonstrates the performance of the FPA-CCDE on robot path planning. The last part of this paper is Section 6, which provides conclusions.

## 2. Preliminary Review

Cross-pollination and self-pollination are the two components of flower pollination. Cross-pollination signifies that pollination occurs among different flowers due to natural wind transmission and animal carrying. Self-pollination relates to the pollination of the same flower by spontaneous diffusion without consequence by biological factors. In the FPA, Yang [25] regards these two pollination methods as global and local searching processes, respectively. Moreover, Yang employs switch probability to realize the conversion between two methods, which can substantially balance the ratio of global and local searches.

Cross-pollination corresponds to the global searching process of the FPA. Let the population size of pollen be *N*, then the position of pollen *i* at iteration t+1 is:

(1) $$x_{i}^{t+1}=x_{i}^{t}+L\cdot \left(x_{i}^{t}-g^{*}\right),$$

where $x_{i}^{t}$ is the pollen *i* at iteration *t*, and $g^{*}$ is the current fittest pollen. *L* denotes the step size of the pollination, which follows a Levy distribution [39].

Self-pollination is in line with the local searching process of the FPA, and the pollen *i* at iteration t+1 is:

(2) $$x_{i}^{t+1}=x_{i}^{t}+\varepsilon \cdot \left(x_{j}^{t}-x_{k}^{t}\right),$$

where $x_{j}^{t}$ and $x_{k}^{t}$ represent two pollen individual positions (different flowers belonging to the same flowering plant) in the population that are distinct from pollen individual *i*, which essentially mimics the constancy of flowers in a finite region and ε follows a uniform distribution in [0,1].

## 3. The Flower Pollination Algorithm Based on Cosine Cross-Generation Differential Evolution (FPA-CCDE)

**Figure 1:**
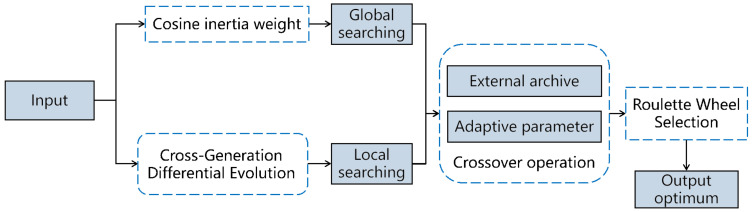
The framework of the FPA-CCDE.

As a novel heuristic algorithm for optimization, the FPA has fewer parameters and can achieve high efficiency. Nonetheless, it is prone to falling into the local optimum, and it has insufficient population richness and search accuracy. In light of this, we propose the FPA-CCDE algorithm. The framework of the FPA-CCDE is shown in Figure 1. The cosine inertia weights are introduced in the global search process to enhance the global search accuracy and balance the searching process. Moreover, the introduction of cross-generation difference in the local search process differential evolution can increase the population’s diversity and the efficiency of iteration. The parameter adaptive adjustment mechanism can increase the effectiveness of searching, and the roulette wheel selection mechanism can help the algorithm jump out of the local optimal in the searching process. More details are given in the following subsections.

### 3.1. Cosine Inertia Weight

The inertia weight in the traditional FPA is a random value. A larger inertia weight can contribute to better global searching. Smaller inertia weight assists to strengthen the local searching process. Notwithstanding, this kind of weight cannot guide the global search direction and even leads to local optimal [40]. Consequently, in the global searching process, to improve global search precision, we design a cosine inertia weight on the basis of the simulated annealing method as

(3) $$\omega =\lambda \left[1-\cos\left(\frac{\pi }{2}\frac{t}{T_{\max}}\sqrt{d_{num}}\right)\right]+\sigma betarnd\left(a,b\right),$$

where λ is the weight adjustment factor, $T_{\max}$ is the maximum number of iterations, *t* is the current iteration, and σ is the inertia adjustment factor. The second term on the right-hand side utilizes the beta distribution to adjust the total inertia weight value. The deviation degree of ω can control the inertia weight value then strengthen the global searching as well as the local searching ability.

On the basis of the cosine inertia weight, the position of pollinate iteration t+1 in global searching can be rewritten as:

(4) $$x_{i}^{t+1}=x_{i}^{t}-k\cdot \left(x_{i}^{t}-x_{cen}^{t}\right)+rand\left(1,d_{num}\right)\cdot U_{\min}^{i}+\left(U_{\max}^{i}-U_{\min}^{i}\right),$$

where $U_{\max}^{i}$ and $U_{\min}^{i}$ are the upper and lower limits of pollen individual values in each dimension, respectively, and $d_{num}$ is the dimension of pollen individual. Here, $rand\left(1,d_{num}\right)$ is a matrix of $1\times d_{num}$, and the values of each column are drawn from a uniform distribution in the range [0,1]. Finally, $x_{cen}^{t}$ refers to the average value of the upper and lower dimensions of each individual.

### 3.2. Cross-Generation Differential Evolution

In the local searching process, we employ cross-generation differential evolution [41] to achieve a balance between convergence speed and diversity, while maintaining a balance between exploration and development.

The cross-generation differential evolution includes two mutation strategies, namely the neighborhood-based cross-generation strategy (NCG) and the population-based cross-generation strategy (PCG). The NCG estimates the promising search direction by analyzing the differences between consecutive generations. It can direct the algorithm toward the optimal person. In the mutation of the PCG, the populations from two generations participate in the search for the optimal individual. Combining the information from two generations can reduce the numerical oscillation of search results and improve the search stability of the FPA in the local searching process. In this paper, to balance the convergence and search ability, we adopt the NCG and the PCG with the same probability during the mutation process. The generation of new individuals from the NCG and the PCG is described in detail following the table.

In the NCG, each individual called parent individual will be mutated, and two neighborhood pools of the parent individuals are used to generate the mutant vector. One neighborhood pool is formed by *T* individuals from a population of the current generation, and the other neighborhood pool consists of *T* individuals from the population of the previous generation.

The selection criteria are listed below. By calculating the Euclidean distance between the parent and other individuals in the population, the nearest *T* individuals are selected to form one neighborhood pool. In accordance with the Euclidean distance, *T* individuals are also picked from the previous generation as the members of the other neighborhood pool.

Utilizing two neighborhood pools for mutation, the new individual generated after mutation can be expressed as:

(5) $$V_{i,g}=x_{rn_{1},g}+F\cdot \left(x_{rn_{1},g}-x_{rn_{2},g-1}\right),$$

where $V_{i,g}$ is the mutant vector, *i* is the index of the parent individual, *g* is the index of the mutation generation, $x_{rn_{1},g}$ is the randomly selected individual in the neighborhood pool of the current generation, and $x_{rn_{2},g-1}$ is the spontaneously selected individual in the parent individual neighborhood pool of the previous generation. Subscript $rn_{1}$ is an integer randomly selected from $\left\{I_{i,g,1},I_{i,g,2},\ldots ,I_{i,g,T}\right\}$, which records the indices of *T* members in the neighborhood pool. Similarly, $rn_{2}$ is randomly selected from $\left\{I_{i,g-1,1},I_{i,g-1,2},\ldots ,I_{i,g-1,T}\right\}$, and the size is 5% of the total population size.

In PCG, the new individual generated after mutation is:

(6) $$V_{i,g}=x_{i,g}+F\cdot \left(x_{rp_{1},g}-x_{rp_{2},g-1}\right),$$

where $x_{i,g}$ is the parent individual, $x_{rp_{1},g}$ is the randomly selected individual in the population of the current generation, individual $x_{rp_{2},g-1}$ is in the population of the previous generation of the parent individual, $rp_{1}$ and $rp_{2}$ are random integers selected from $\left\{1,2,\ldots ,N\right\}$, and *N* is population size.

### 3.3. External Archiving Mechanism

After the searching process, to select high-quality solutions in the neighborhood pool, an external archiving mechanism is proposed.

Population diversity can be increased by establishing external archives and requiring individuals within those archives to direct the search process. After completing differential evolution, the fitness value of the resulting offspring $u_{i}$ is compared with the fitness value of the parent $x_{i}$. If the fitness value is smaller, then the offspring $u_{i}$ replaces $x_{i}$. Otherwise, T−1 individuals whose fitness value is greater than $x_{i}$ are selected and stored in an archive. External archiving can increase the richness of the population and avoid the search process from falling into a locally optimal solution. In addition, it can direct individuals in their search for potential subregions with high-quality solution options. After completing the search iteration, if the number of solutions contained in the external archive exceeds the threshold value $N_{p}$ individuals are randomly removed from the archive to keep the number within $N_{P}$.

### 3.4. Parameter Adaptive Adjustment Mechanism

Since NCG and PCG are highly sensitive to scaling factors and crossover probability CR, the value of these two parameters will affect the sear process [41,42]. If the scaling factor is small, the difference vector will generate a small oscillation, and the richness of the population decreases. If *F* is large, it will increase the blindness of the searching process, and the convergence velocity slows down. CR determines the probability of crossover between the parent individual and mutation vector in balancing local and global search processes. If CR is large, the population diversity and convergence speed will be increased. For distinct stages of the search process, it is essential to formulate distinct parameters.The parameters setting should meet the following requirements: (1) Select appropriate parameters for a specific region in the target scope; (2) Eliminate inappropriate parameters; (3) Reduce the probability of complete convergence of parameters.

With every iteration, the $F_{i,g}$ of each individual $x_{i}$ is determined according to the Cauchy distribution, which can be expressed as:

(7) $$F_{i,g}=randc_{i}\left(\mu_{F},0.1\right).$$

If $F_{i}\geq 1$, set $F_{i}$ to 1. If $F_{i}\leq 0$, $F_{i}$ will be generated again according to (7). The initial value is 0.5, and the updated formula is as follows:

(8) $$\mu_{F}=\frac{1}{2}\cdot \mu_{F}+\frac{1}{2}\cdot mean_{L}\left(S_{F}\right).$$

(9) $$mean_{L}\left(S_{F}\right)=\frac{\sum_{F\in S_{F}}F^{2}}{\sum_{F\in S_{F}}F^{}}.$$

$S_{F}$ is the set in the archive of $F_{i}$ of the successful individuals at iteration *t* and is used to denote the Lehmer of all successful individuals in the population.

The generation mainly includes two operation processes. The Cauchy distribution is advantageous for the diversification of mutation factors and avoids the drawback of premature convergence in the differential evolution strategy searching process. Likewise, the Lehmer mean will give more weight to the scaling factor of the better solution. The mutation factor information of the better solution is transmitted to improve the optimization efficiency, and the larger scaling factor occupies a greater weight in the calculation of $\mu_{F}$. It can take optimization precision and algorithm operation efficiency into account.

The crossover is probably randomly generated for utility. The crossover probability updates as:

(10) $$CR_{i,g}=\left\{\begin{array}{c} CR_{\min}+\frac{\left(CR_{\max}-CR_{\min}\right)\;\left(f_{i}-f_{\min}\right)}{f_{\max}-f_{\min}}*\frac{f_{4}-f_{2}}{f_{3}-f_{1}},{}^{}f_{i}\geq \bar{f},\\ CR_{\min},f_{i}<\bar{f}, \end{array}\right.$$

where $CR_{i,g}$ is the average fitness for all individuals within the current population. Randomly selecting four individuals, $x_{p1}$, $x_{p2}$, $x_{p3}$, $x_{p4},$ from the external archived population, their fitness values are $f_{1}$, $f_{2}$, $f_{3}$, $f_{4}$, respectively, and $f_{4}>f_{3}>f_{2}>f_{1}$.

After completing the local search, the subsequent cross-selection procedure is also carried out as follows:

(11) $$U_{i,g}=\left\{\begin{array}{c} V_{i,g}\begin{array}{ccc} , & if & rand<CR_{i,g}, \end{array}\\ x_{i,g}\begin{array}{cccc} , & if & rand\geq CR_{i,g}, & \end{array} \end{array}\right.$$

where $U_{i,g}$ is the trailing vector and rand is derived from a uniform distribution in the range [0,1]. After completing the pollen variation and cross-selection of the population’s individuals, the selection is carried out:

(12) $$X_{i,g+1}=\left\{\begin{array}{c} U_{i,g}\begin{array}{cc} , & if \end{array}\;\;f\left(U_{i,g}\right)\leq f\left(X_{i,g}\right).\\ X_{i,g}\begin{array}{cc} , & if \end{array}\;\;f\left(U_{i,g}\right)>f\left(X_{i,g}\right). \end{array}\right.$$

### 3.5. Cross-Generation Roulette Wheel Selection

Heuristic optimization algorithms predominantly applied roulette wheel selection to jump out of the local optimal in the searching process [13,43]. After completing a search iteration for all individuals, we select *T* pollen individuals with the smallest fitness value and then randomly select *T* pollen individuals from the remaining individuals in the population. For the individual $x_{i}^{t+1}\left(i=1,2,\ldots 2T\right)$, we design a cross-generation roulette wheel selection mechanism to reduce the likelihood of algorithmic failure falling into a local solution.

We select *T* individuals which include parent individuals of the current population and individuals of the previous generation (we recommend α=0.7T and β=0.3T) to form a roulette wheel pool to engage in the process of roulette wheel selection. The probability of each pollen individual being selected in the roulette pool is:

(13) $$p_{i}=\frac{wig_{i}}{\sum_{i=1}^{2T}wig_{i}}.$$

For pollen individuals *i*, $wig_{i}$ is the mapping weight of the fitness value. The cross-generation roulette wheel selection strategy selects appropriate parameters in the target scope. Consequently, it can eliminate inappropriate parameters and reduce the probability of complete convergence of parameters. The specific actions are broken down into the following steps.

Step 1: Sort the pollen individuals in the parent population in accordance with the fitness values.

Step 2: Select the top *T* pollen individuals to form a subpopulation $P_{t}$.

Step 3: Randomly select β remaining individuals from the parent population.

Step 4: Randomly select α individuals from the current population.

Step 5: Combine subpopulations $P_{t}$ with selected individuals from the current population to form a roulette wheel selection pool.

Step 6: The individuals in the roulette pool perform the roulette process according to (13).

On the basis of the above analysis in this section, the pseudo-code of the FPA-CCDE can be summarized in Algorithm 1.
**Algorithm 1** Flower Pollination Algorithm Based on Cosine Cross-Generation Differential Evolution (FPA-CCDE)1:Initialize the pollen population size *N* and optimal individual $g^{*}$.2:Set the maximum criterion $T_{\max}$, and select initial crossover probability $CR_{0}$ and initial scaling factor $F_{0}$ respectively from $\left[CR_{\min},CR_{\max}\right]$ and $\left[F_{\min},F_{\max}\right]$.3:Randomly generate switch probabilities rand1 and rand2.4:**for** each individual in current population **do**5: Calculate $F_{i,g}$ according to (7).6: **if** rand1<P **then**7:   **if** rand2<0.5 **then**8:    Updating the current pollen position using PCG differential evolution.9:   **else**10:    Updating the current pollen position using NCG differential evolution.11:    Crossover and selecting operation for $x_{i+1}$ according to formulas (10) and (11).12:   **end if**13: **else**14:  For cross-pollination, formula (4) is used to complete the global search process and update the current pollen position.15:  Select the updated pollen individuals to perform mutation and crossover operations according to formulas (11) and (12).16: **end if**17: Generate $x_{i}^{p}$ using cross-generation Roulette Wheel Selection Mechanism.18: Compare $x_{i}^{p}$ with $x_{i+1}$ and replace $x_{i+1}$ if $x_{i}^{p}$ has a better fitness value.19: Compare $x_{i+1}$ with $x_{i}$ and replace $x_{i}$ if $x_{i+1}$ has a better fitness value.20: Compare $x_{i+1}$ with $g^{*}$ and replace $g^{*}$ if $x_{i+1}$ has a better fitness value.21:**end for**22:Repeat line 3 to line 19 until the $T_{\max}$ is satisfied.

## 4. Evaluation forthe FPA-CCDE

To verify the optimization performance of the proposed FPA-CCDE and the operation efficiency of the algorithm, in this section, 32 sets of standard test functions [41,42] were chosen to test the FPA-CCDE, and particle swarm optimization algorithm (PSO) was used to conduct research and comparisons with the algorithm, the pollination algorithm (FPA), the artificial bee colony algorithm (ABC), the genetic algorithm (GA), and the spider clustering algorithm (SSO) in the identical testing setting. In accordance with Yang Xin’s article [33], each algorithm is run independently 30 times, and the algorithm is suitable for most applications on the condition that the transition probability P=0.8. The pollen crossover probability $CR_{0}=0.15$ is randomly initialized, the population size $N_{p}=300$ is set for all comparison algorithms, and the maximum number of iterations is set to $T_{\max}=2500$. The parameters setting for Algorithm 1 are given in Table 1. The selected test functions mainly include three types, namely, the low-dimensional test function (dimension less than 10) in Table 2, the high-dimensional test function (dimension more than 10) in Table 3, and the extensible test function [43]. Among them, F10 is a low-dimensional multipeak function, which makes it simple for the algorithm to find the local optimal solution during the search. Most low-dimensional functions tend to oscillate violently throughout the search process.

### 4.1. Performance Comparison on Low-Dimensional Benchmark Functions

The low-dimensional benchmark functions are frequently utilized in numerous performance evaluations of heuristic optimization algorithms and can generate a mass of local optimum solutions. These functions are continuous or discontinuous, convex or non-convex, and unimodal or multimodal.

The fitness value and standard deviation on benchmark functions of divergent algorithms are displayed in Table 2, with EF fixed at 1000. The smaller the fitness value and standard deviation are, the higher the optimization accuracy and stability will be. For instance, the fitness value and standard deviation of the FPA-CCDE on function F2 are smaller than that of other algorithms, so the optimization accuracy and stability of the FPA-CCDE are the best. It can be observed from Table 2 that compared with the FPA, the FPA-CCDE appears to be better in 15 functions (F2–F4, F7, F8, F14, F17, F18, F20, F22, F28, F30–F32, F35, F36). The standard deviation of the FPA-CCDE has undergone significant enhancements in 18 functions. The results indicate that the FPA-CCDE greatly enhances the optimization accuracy in the searching process. Moreover, the smaller standard deviation values demonstrate that the FPA-CCDE has smaller numerical oscillation and higher stability.

In comparison to ABC, the FPA-CCDE achieves considerably better fitness value in 8 functions and obtains similar fitness value in 10 functions. For standard deviation, the FPA-CCDE outperforms ABC in 12 functions. In general, the optimization accuracy and stability of the FPA-CCDE are fairly superior to ABC. In the listed functions, the FPA-CCDE surpasses PSO in F14, F17, F30, and F35-F36 on fitness value, and it is similar to PSO in 12 functions. For standard deviation, the FPA-CCDE wins in 7 (F16–F18, F22, F35–F36) functions, and ties in 4 (F3–F4, F7, F28) functions. For functions F2, F5, F8, and F31–F32, although PSO achieves a smaller standard deviation, it is not significantly different from the FPA-CCDE at the level α=0.05. In 16 functions, the FPA-CCDE outperforms the GA in optimization accuracy. Regarding standard deviation, the FPA-CCDE only loses in F5, and it can find smaller fitness value than the GA in most functions. Compared with SSO, the FPA-CCDE can provide better or similar fitness value in 18 functions.

**Table 2:** Experimental results of benchmark function with low dimension.

Functions	FPA-CCDE	FPA [14]	ABC [15]	PSO [16]	GA [17]	SSO [18]
F2	**3.1021 × 10${}^{-15}$** **(4.3388 × 10${}^{-15}$)**	4.1451 × 10${}^{-3}$ (7.0754 × 10${}^{-3}$)	6.8256 × 10${}^{-10}$ (1.3239 × 10${}^{-9}$)	**0.0000 × 10${}^{0}$** **(0.0000 × 10${}^{0}$)**	7.8197 × 10${}^{-2}$ (1.6845 × 10${}^{-1}$)	1.5299 × 10${}^{-7}$ (1.7006 × 10${}^{-7}$)
F3	**0.0000 × 10${}^{0}$** **(0.0000 × 10${}^{0}$)**	2.2574 × 10${}^{-1}$ (2.2744 × 10${}^{-1}$)	**0.0000 × 10${}^{0}$** **(0.0000 × 10${}^{0}$)**	**0.0000 × 10${}^{0}$** **(0.0000 × 10${}^{0}$)**	6.2051 × 10${}^{-1}$ (2.9506 × 10${}^{-1}$)	1.9375 × 10${}^{-6}$ (1.4281 × 10${}^{-6}$)
F4	**0.0000 × 10${}^{0}$** **(0.0000 × 10${}^{0}$)**	1.7376 × 10${}^{0}$ (1.3740 × 10${}^{0}$)	2.8247 × 10${}^{-1}$ (1.7454 × 10${}^{-1}$)	**0.0000 × 10${}^{0}$** **(0.0000 × 10${}^{0}$)**	4.7987 × 10${}^{1}$ (3.1709 × 10${}^{1}$)	1.2790 × 10${}^{-3}$ (5.9245 × 10${}^{-4}$)
F5	**−2.0626 × 10${}^{0}$** **(3.1346 × 10${}^{-12}$)**	**−2.0626 × 10${}^{0}$** **(1.8779 × 10${}^{-5}$)**	**−2.0626 × 10${}^{0}$** **(9.0649 × 10${}^{-16}$)**	**−2.0626 × 10${}^{0}$** **(9.0649 × 10${}^{-16}$)**	−2.0625 × 10${}^{0}$ (1.3597 × 10${}^{-15}$)	**−2.0626 × 10${}^{0}$** **(1.2666 × 10${}^{-8}$)**
F7	**−1.0000 × 10${}^{0}$** **(0.0000 × 10${}^{0}$)**	−9.5125 × 10${}^{-1}$ (2.7284 × 10${}^{-2}$)	**−1.0000 × 10${}^{0}$** **(0.0000 × 10${}^{0}$)**	**−1.0000 × 10${}^{0}$** **(0.0000 × 10${}^{0}$)**	−9.5125 × 10${}^{-1}$ (1.1331 × 10${}^{-16}$)	−9.9999 × 10${}^{-1}$ (4.6850 × 10${}^{-6}$)
F8	**−1.0000 × 10${}^{0}$** **(0.0000 × 10${}^{0}$)**	−7.9984 × 10${}^{-1}$ (3.5024 × 10${}^{-1}$)	−9.9999 × 10${}^{-1}$ (8.4234 × 10${}^{-6}$)	**−1.0000 × 10${}^{0}$** **(0.0000 × 10${}^{0}$)**	−5.4542 × 10${}^{-1}$ (4.9345 × 10${}^{-1}$)	**−1.0000 × 10${}^{0}$** **(2.1452 × 10${}^{-7}$)**
F14	**3.0000 × 10${}^{0}$** **(2.0121 × 10${}^{-15}$)**	3.0024 × 10${}^{0}$ (3.0070 × 10${}^{-3}$)	**3.0000 × 10${}^{0}$** **(1.8198 × 10${}^{-7}$)**	**3.0000 × 10${}^{0}$** **(1.2128 × 10${}^{-15}$)**	3.9060 × 10${}^{0}$ (1.0226 × 10${}^{0}$)	3.00004 × 10${}^{0}$ (3.6979 × 10${}^{-5}$)
F16	**−3.8627 × 10${}^{0}$** **(1.8995 × 10${}^{-15}$)**	**−3.8627 × 10${}^{0}$** **(7.5327 × 10${}^{-5}$)**	**−3.8627 × 10${}^{0}$** **(2.7194 × 10${}^{-15}$)**	**−3.8627 × 10${}^{0}$** **(2.6691 × 10${}^{-15}$)**	**−3.8627 × 10${}^{0}$** **(1.8206 × 10${}^{-5}$)**	**−3.8627 × 10${}^{0}$** **(4.2692 × 10${}^{-5}$)**
F17	**−3.0679 × 10${}^{0}$** **(3.1312 × 10${}^{-18}$)**	−3.0155 × 10${}^{0}$ (2.9497 × 10${}^{-2}$)	**−3.0424 × 10${}^{0}$** **(9.0649 × 10${}^{-16}$)**	−3.0031 × 10${}^{0}$ (3.0102 × 10${}^{-2}$)	−3.0113 × 10${}^{0}$ (2.8284 × 10${}^{-2}$)	−3.0305 × 10${}^{0}$ (2.2595 × 10${}^{-2}$)
F18	**−1.9208 × 10${}^{1}$** **(5.4266 × 10${}^{-15}$)**	−1.9207 × 10${}^{1}$ (1.4184 × 10${}^{-3}$)	**−1.9208 × 10${}^{1}$** **(7.7768 × 10${}^{-15}$)**	**−1.9208 × 10${}^{1}$** **(6.1960 × 10${}^{-15}$)**	−1.9207 × 10${}^{1}$ (5.2294 × 10${}^{-15}$)	−1.9208 × 10${}^{1}$ (8.5032 × 10${}^{-7}$)
F20	**−1.8013 × 10${}^{0}$** **(4.2850 × 10${}^{-16}$)**	−1.8011 × 10${}^{0}$ (2.7689 × 10${}^{-4}$)	**−1.8013 × 10${}^{0}$** **(6.7987 × 10${}^{-16}$)**	**−1.8013 × 10${}^{0}$** **(6.7987 × 10${}^{-16}$)**	−1.8011 × 10${}^{0}$ (8.7772 × 10${}^{-8}$)	**−1.8013 × 10${}^{0}$** **(2.0978 × 10${}^{-6}$)**
F22	**2.9197 × 10${}^{-6}$** **(2.5958 × 10${}^{-6}$)**	1.2187 × 10${}^{1}$ (6.1220 × 10${}^{0}$)	1.8688 × 10${}^{-2}$ (1.7140 × 10${}^{-2}$)	1.4393 × 10${}^{-1}$ (2.6987 × 10${}^{-1}$)	4.4791 × 10${}^{6}$ (9.9631 × 10${}^{6}$)	5.7462 × 10${}^{-2}$ (1.6986 × 10${}^{-1}$)
F28	**0.0000 × 10${}^{0}$** **(0.0000 × 10${}^{0}$)**	5.0174 × 10${}^{-3}$ (5.2430 × 10${}^{-3}$)	1.5375 × 10${}^{-9}$ (3.4115 × 10${}^{-9}$)	**0.0000 × 10${}^{0}$** **(0.0000 × 10${}^{0}$)**	1.0890 × 10${}^{-3}$ (1.7702 × 10${}^{-3}$)	3.8345 × 10${}^{-10}$ (1.0001 × 10${}^{-10}$)
F30	**−1.0536 × 10${}^{1}$** **(1.1934 × 10${}^{-14}$)**	−7.2096 × 10${}^{0}$ (3.4956 × 10${}^{0}$)	−1.0536 × 10${}^{1}$ (6.2565 × 10${}^{-5}$)	5.8020 × 10${}^{0}$ (3.3779 × 10${}^{0}$)	−6.5184 × 10${}^{0}$ (3.1127 × 10${}^{0}$)	−1.0533 × 10${}^{1}$ (1.5542 × 10${}^{-3}$)
F31	**−1.0316 × 10${}^{0}$** **(2.1531 × 10${}^{-11}$)**	−1.0319 × 10${}^{0}$ (3.8595 × 10${}^{-5}$)	**−1.0316 × 10${}^{0}$** **(2.2662 × 10${}^{-16}$)**	**−1.0316 × 10${}^{0}$** **(2.2662 × 10${}^{-16}$)**	−1.0302 × 10${}^{0}$ (1.2697 × 10${}^{-3}$)	−1.0316 × 10${}^{0}$ (1.1275 × 10${}^{-6}$)
F32	**−1.9410 × 10${}^{0}$** **(3.2685 × 10${}^{-9}$)**	**−1.9410 × 10${}^{0}$** **(1.2658 × 10${}^{-5}$)**	**−1.9410 × 10${}^{0}$** **(4.5324 × 10${}^{-16}$)**	**−1.9410 × 10${}^{0}$** **(4.9650 × 10${}^{-16}$)**	**−1.9410 × 10${}^{0}$** **(1.0748 × 10${}^{-6}$)**	**−1.9410 × 10${}^{0}$** **(8.4117 × 10${}^{-6}$)**
F35	**4.5477 × 10${}^{-87}$** **(2.8866 × 10${}^{-96}$)**	7.0076 × 10${}^{-5}$ (1.3636 × 10${}^{-4}$)	1.8304 × 10${}^{-18}$ (1.5938 × 10${}^{-18}$)	4.4158 × 10${}^{-72}$ (2.2079 × 10${}^{-71}$)	1.3759 × 10${}^{-4}$ (1.3743 × 10${}^{-4}$)	1.40547 × 10${}^{-7}$ (1.24796 × 10${}^{-7}$)
F36	**−1.5198 × 10${}^{3}$** **(2.1675 × 10${}^{-1}$)**	3.5801 × 10${}^{4}$ (1.3014 × 10${}^{4}$)	−1.3389 × 10${}^{3}$ (1.1533 × 10${}^{2}$)	−9.2442 × 10${}^{2}$ (5.3658 × 10${}^{2}$)	1.4270 × 10${}^{5}$ (4.1396 × 10${}^{4}$)	−1.4922 × 10${}^{3}$ (5.4167 × 10${}^{1}$)
+/=/−		15/3/0	8/10/0	5/12/1	16/2/0	14/4/0

The outcomes demonstrate that for the low-dimensional test function, the FPA-CCDE greatly improves the reliability of the optimization accuracy in the searching process with smaller numerical oscillation and higher stability.

### 4.2. Performance Comparison on High-Dimensional Benchmark Functions

To verify the stability of the FPA-CCDE, we test it on high-dimensional benchmark functions with 30 dimensions. Such capabilities have a huge number of locally optimal solutions, which may hamper the whole optimization process. The corresponding experiment results are shown in Table 3.

We can see that the optimization precision of the FPA-CCDE is superior to that of the FPA for all functions. The FPA-CCDE achieves better solutions and mean error values than ABC in all functions except for F6. Moreover, the FPA-CCDE can obtain better solutions and error values than PSO, the GA ,and SSO in all functions.

**Table 3:** Experimental results of benchmark function with high dimension.

Functions	FPA-CCDE	FPA	ABC	PSO	GA	SSO
F1	**9.4147 × $10^{-15}$** **(1.3831 × $10^{-14}$)**	1.5080 × $10^{1}$ (9.7566 × $10^{-1}$)	5.7545 × $10^{-6}$ (2.5028 × $10^{-6}$)	4.1650 × $10^{0}$ (6.5479 × $10^{-1}$)	1.6527 × $10^{1}$ (5.8660 × $10^{-1}$)	2.9000 × $10^{-1}$ (3.3065 × $10^{-2}$)
F6	1.5782 × $10^{-2}$ (1.2026 × $10^{-2}$)	3.2860 × $10^{4}$ (1.8733 × $10^{4}$)	**1.0434 × $10^{-2}$** **(5.7614 × $10^{-3}$)**	6.9923 × $10^{0}$ (3.3423 × $10^{0}$)	3.5530 × $10^{5}$ (1.0824 × $10^{5}$)	2.5406 × $10^{0}$ (9.0102 × $10^{-1}$)
F15	**0.0000 × $10^{0}$** **(0.0000 × $10^{0}$)**	7.8836 × $10^{1}$ (2.0933 × $10^{1}$)	5.0017 × $10^{-4}$ (2.5007 × $10^{-3}$)	8.5954 × $10^{-1}$ (4.1484 × $10^{-1}$)	1.8969 × $10^{2}$ (2.5099 × $10^{1}$)	1.8620 × $10^{-2}$ (1.2621 × $10^{-2}$)
F19	**1.4998 × $10^{-32}$** **(2.5761 × $10^{-29}$)**	3.5660 × $10^{1}$ (8.0118 × $10^{0}$)	4.8711 × $10^{-14}$ (3.3297 × $10^{-14}$)	1.5369 × $10^{0}$ (9.8456 × $10^{-1}$)	7.0606 × $10^{1}$ (9.2977 × $10^{0}$)	1.4748 × $10^{-1}$ (2.3450 × $10^{-1}$)
F21	**7.8109 × $10^{-14}$** **(1.9111 × $10^{-13}$)**	1.1668 × $10^{1}$ (2.9367 × $10^{-1}$)	1.1511 × $10^{0}$ (2.0025 × $10^{-1}$)	6.2434 × $10^{0}$ (8.4791 × $10^{-1}$)	1.1301 × $10^{1}$ (4.5514 × $10^{-1}$)	3.5535 × $10^{0}$ (8.1652 × $10^{-1}$)
F23	**1.7355 × $10^{-73}$** **(3.0057 × $10^{-73}$)**	4.9245 × $10^{2}$ (1.8600 × $10^{2}$)	4.3686 × $10^{-2}$ (1.3277 × $10^{-2}$)	4.9038 × $10^{0}$ (3.2106 × $10^{0}$)	4.0988 × $10^{3}$ (1.0626 × $10^{3}$)	1.6126 × $10^{0}$ (5.5078 × $10^{-1}$)
F24	**−1.4344 ×$10^{4}$** **(3.6431 × $10^{3}$)**	−4.9410 ×$10^{3}$ (3.8186 ×$10^{2}$)	−1.2208 ×$10^{4}$ (2.2864 ×$10^{2}$)	−3.2450 ×$10^{3}$ (3.5118 ×$10^{2}$)	−5.6765 ×$10^{3}$ (1.8977 ×$10^{3}$)	−7.9893 ×$10^{3}$ (8.4372 ×$10^{2}$)
F25	**1.1137 × $10^{-10}$** **(3.0381 × $10^{-10}$)**	2.3523 × $10^{2}$ (1.6087 × $10^{1}$)	1.8038 × $10^{-2}$ (7.4305 × $10^{-2}$)	3.9470 × $10^{1}$ (8.9127 × $10^{0}$)	2.4371 × $10^{2}$ (1.7642 × $10^{1}$)	4.7235 × $10^{1}$ (1.0067 × $10^{1}$)
F26	**0.0000 × $10^{0}$** **(0.00000 × $10^{0}$)**	2.3426 × $10^{4}$ (1.2743 × $10^{4}$)	2.7476 × $10^{-1}$ (2.0968 × $10^{-1}$)	1.8593 × $10^{2}$ (6.6331 × $10^{1}$)	2.5259 × $10^{5}$ (9.1306 × $10^{4}$)	6.7862 × $10^{1}$ (3.6053 × $10^{1}$)
F27	**9.5293 × $10^{-78}$** **(3.1604 × $10^{-77}$)**	4.7212 × $10^{4}$ (1.3112 × $10^{4}$)	1.6450 × $10^{-}$11 (1.4457 × $10^{-}$11)	3.3784 × $10^{0}$ (2.8782 × $10^{0}$)	1.2034 × $10^{5}$ (2.3265 × $10^{4}$)	1.1852 × $10^{0}$ (2.7957 × $10^{-1}$)
F29	**1.2451 × $10^{-4}$** **(3.7184 × $10^{-6}$)**	6.9380 × $10^{3}$ (4.9156 × $10^{2}$)	1.6629 × $10^{2}$ (1.1654 × $10^{2}$)	9.2941 × $10^{3}$ (3.6015 × $10^{2}$)	5.9258 × $10^{3}$ (4.1609 × $10^{2}$)	6.7862 × $10^{1}$ (3.6053 × $10^{1}$)
F33	**0.0000 × $10^{0}$** **(0.0000 × $10^{0}$)**	8.9432 × $10^{3}$ (2.3352 × $10^{3}$)	**0.0000 × $10^{0}$** **(0.0000 × $10^{0}$)**	6.5480 × $10^{1}$ (4.5312 × $10^{1}$)	2.2026 × $10^{4}$ (2.8696 × $10^{3}$)	2.4000 × $10^{-1}$ (4.3589 × $10^{-1}$)
F34	**6.2388 × $10^{-28}$** **(1.1777 × $10^{-27}$)**	1.3207 × $10^{1}$ (1.5938 × $10^{0}$)	3.0464 × $10^{-1}$ (8.7926 × $10^{-2}$)	2.0137 × $10^{0}$ (1.0508 × $10^{0}$)	1.4142 × $10^{1}$ (1.2205 × $10^{0}$)	2.9222 × $10^{0}$ (5.1325 × $10^{-1}$)
F37	**1.6338 × $10^{-2}$** **(2.1111 × $10^{-2}$)**	1.2515 × $10^{2}$ (1.2515 × $10^{2}$)	2.4360 × $10^{2}$ (2.6737 × $10^{1}$)	9.5543 × $10^{-1}$ (4.8483 × $10^{-1}$)	2.5296 × $10^{8}$ (4.6531 × $10^{8}$)	5.0225 × $10^{-1}$ (1.1329 × $10^{-1}$)
+/=/−		14/0/0	12/1/1	14/0/0	14/0/0	14/0/0

### 4.3. Performance Comparison on Scalable Benchmark Functions

To further verify the optimization effect of the FPA-CCDE under scalable dimensions of benchmark functions, we select F9 as the test function. F9 has a global minimum solution $\left(0,0,\ldots 0\right){}_{n}$, which is surrounded by a massive local minimum optimum with identical function values. Meanwhile, maximum solutions exist between the global minimum and local minimum solutions. Hence, its global convergence speed is poor, and it is simple to fall into local optimum.

The fitness values of optimum solutions and standard deviations of function F9 are summarized in Table 4. It can be observed that in the case of low-dimensional test functions, the optimum solution fitness values of algorithms, such as ABC and PSO, are close to the FPA-CCDE. This is because in this case, the number of locally optimal solutions is small, and it is simple to deviate from the local optimal solutions. Nevertheless, the increase of the dimension of variables will generate more local optimal solutions, and most heuristic optimization algorithms will lack searching directions, contributing to an unbalanced local–global searching process. In this situation, the superiority of the FPA-CCDE improves search accuracy and guides the search process. In Table 4, we can see that the optimum solution fitness values and standard deviations of the FPA-CCDE are substantially superior to other algorithmic methods.

**Figure 2:**
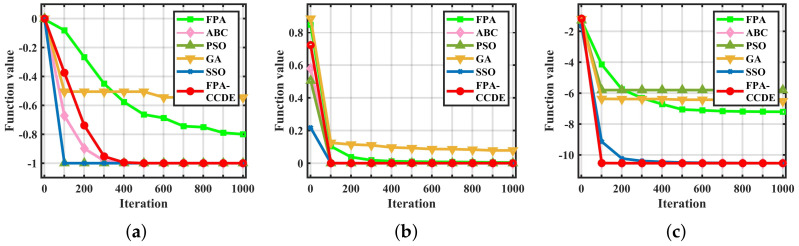
Fitness value graphs of benchmark functions: (**a**) the fitness value of F2; (**b**) the fitness value of F19; (**c**) the fitness value of F30.

**Figure 3:**
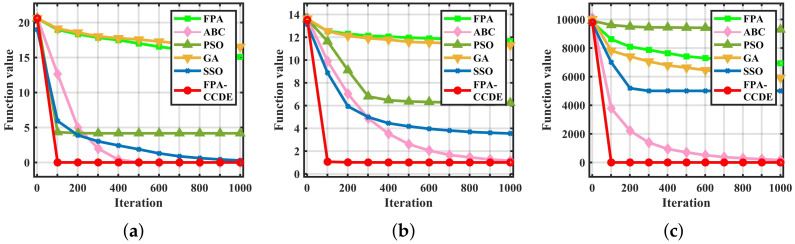
Fitness value graphs of benchmark functions: (**a**) the fitness value of F21; (**b**) the fitness value of F24; (**c**) the fitness value of F29.

**Figure 4:**
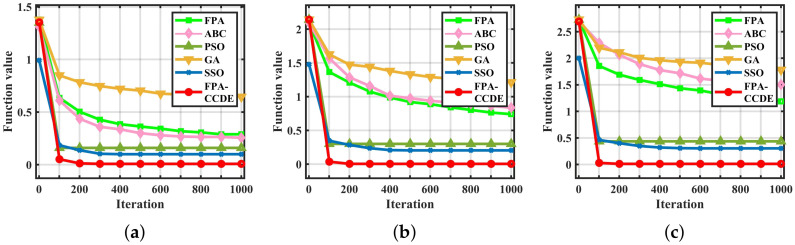
Fitness value graphs on F9: (**a**) the fitness value of F9 with 10 dimensions; (**b**) the fitness value of F9 with 20 dimensions; (**c**) the fitness value of F9 with 30 dimensions.

The iterations to obtain the fitness value of the optimal solution can quantitatively demonstrate convergence and searching efficiency. The experiment results are depicted in Figure 2, Figure 3 and Figure 4. We continue to select the three types of benchmark functions listed above for comparison. In Figure 2, it is obvious that for F2 the iterations required by the GA and the FPA are much higher than other algorithms, and the iterations of the FPA-CCDE are close to ABC, PSO, and SSO, which have better searching efficiency in Figure 2. For another two functions, the iterations required by the FPA-CCDE are slightly less than other algorithms. In Figure 3, although ABC is close to the FPA-CCDE on optimal solution fitness value, the FPA-CCDE outperforms ABC and other algorithms regarding iterations.

Figure 4 demonstrates the fitness values of all algorithms on F9. On the condition of dealing with low-dimensional variables, the optimum fitness value and iteration number of some algorithms are close to that of the FPA-CCDE, respectively. With the increase of variable dimension, the amount of locally optimal solutions increases sharply, and the FPA-CCDE performs better than other algorithms on the fitness value of the optimal solution.

**Figure 5:**
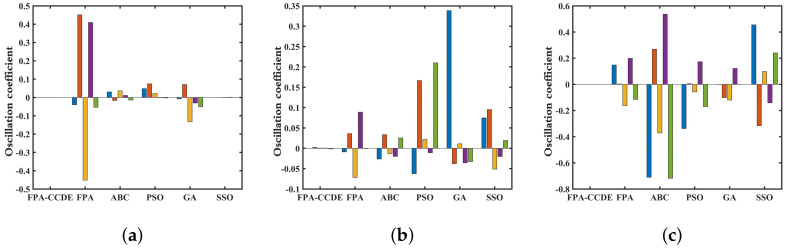
Stability of different algorithms: (**a**) stability on F36; (**b**) stability on F24; (**a**) stability on F25.

With the majority of low-dimensional functions, the algorithm will oscillate violently. Consequently, the stability of the algorithm can be characterized by oscillation. On the condition that the number of optimization iterations of each algorithm is 50, 100, 150, 200, and 250, the fluctuation of accuracy between the optimal solutions obtained through every algorithm is adopted as the performance index of the optimization stability of the algorithm. Each algorithm’s volatility varies concerning specific test functions (F36, F24, F25) and is displayed in Figure 5.

Under the low-dimensional test function (F36), each algorithm’s fluctuation range is relatively stable. As the dimension of the decision variable of the test function increases (F24, F25), the complexity of optimizing the solution space, and the oscillation amplitude of the algorithm increases. In comparison with the other algorithms, the overall oscillation amplitude of the FPA-CCDE is relatively small and has excellent stability.

## 5. Application of the FPA-CCDE in Inspection Robot Path Planning

To evaluate the performance of the FPA-CCDE in a practical scenario, we use it to solve the path planning problem for the inspection robot. The goal of the path planning is to design a motion track in the workplace in accordance with optimization constraints (e.g, minimum energy cost, shortest motion path, minimum duration cost).

In this work, when planning routes, we take into account the following five constraints. The first one is the moving distance limitation. There is a straight-line distance before each turn of the robot for error correction, and the minimum straight-line distance will affect the robot track. If dividing the path into segments, each segment should be greater than the minimum distance limitation. The total distance of segments follows the limitation:

(14) $$\sum_{i=1}^{D+1}\left|\right|P_{i-1}P_{i}\left|\right|\leq L_{\max},$$

where $\left|\right|P_{i-1}P_{i}\left|\right|$ is the distance of a segment, $P_{0}$ is the starting point, $P_{D+1}$ is the endpoint, and $L_{\max}$ is the longest path limit.

The second constraint is the turning angle limitation. The robot has a turning radius between two adjacent segments:

(15) $$\frac{\left(P_{i-1}P_{i}\right)\cdot \left(P_{i}P_{i+1}\right)}{\left|\right|P_{i-1}P_{i}\left|\right|\cdot \left|\right|P_{i}P_{i+1}\left|\right|}\geq \cos\theta_{\max},$$

where $\theta_{\max}$ is the maximum turning angle.

The third constraint is the minimum obstacle distance limitation that guarantees obstacle avoidance. Subsequently, let the current position and direction of a robot be $s_{i}={\left[x_{i},y_{i},\theta_{j}\right]}^{T}$, where $\left(x_{i},y_{i}\right)$ is the position of the robot, $\theta_{j}$ is the steering angle, and the minimum Euclidean distance of the obstacle ϑ to the robot *i* is $d(s_{i},\vartheta )$. Let $d_{\min}$ be the shortest distance that must exist between the robot and the obstruction for there to be no risk of collision between the two. The constraint is expressed as:

(16) $$\begin{array}{c} \vartheta_{i}\left(s_{i}\right)={\left[d\left(s_{i},\vartheta_{1}\right),d\left(s_{i},\vartheta_{2}\right),\ldots ,d\left(s_{i},\vartheta_{R}\right)\right]}^{T}-{\left[d_{\min},d_{\min},\ldots ,d_{\min}\right]}^{T}\geq 0. \end{array}$$

The last two constraints are the velocity and acceleration of the robot. The linear velocity $v_{vi}$, angular velocity $v_{\omega i}$, linear acceleration $a_{vi}$ and angular acceleration $a_{\omega i}$ are defined as:
  (17) $$v_{vi}=\Delta T_{i}^{-1}{\left[x_{i+1}-x_{i},y_{i+1}-y_{i}\right]}^{T},v_{\omega i}=\Delta T_{i}^{-1}\left(\theta_{i+1}-\theta_{i}\right).$$
  (18) $$a_{vi}=\frac{2(v_{v,i+1}-v_{vi})}{\Delta T_{i}+\Delta T_{i+1}},a_{\omega i}=\frac{2(v_{\omega ,i+1}-v_{\omega i})}{\Delta T_{i}+\Delta T_{i+1}}.$$
  Let the maximum linear velocity be $v_{\max}$, the maximum angular velocity be $\omega_{\max}$, the maximum linear acceleration be $a_{\max}$, and the maximum angular acceleration be $\varphi_{\max}$. The limitations can be stated in the following manner:
  (19) $$v_{i}\left(s_{i+1},s_{i},\Delta T_{i}\right)={\left[v_{\max}-\left|v_{vi}\right|,\omega_{\max}-\left|v_{\omega i}\right|\right]}^{T}\geq 0.$$
  (20) $$\begin{array}{c} a_{i}\left(s_{i+2},s_{i+2},\Delta T_{i+1},\Delta T_{i}\right)={\left[a_{\max}-\left|a_{vi}\right|,\varphi_{\max}-\left|a_{\omega i}\right|\right]}^{T}\geq 0. \end{array}$$
  On the basis of the above constraints, we consider four costs, including energy cost, steering cost, threat attacking cost, and time cost.

The energy cost is proportional to the robot’s speed as well as the distance it travels, and this relationship can be expressed as:

(21) $$cost_{e}=\alpha \cdot \frac{v}{3}\cdot l_{i},$$

where α is the consumption factor, *v* is the speed of the robot, and $l_{i}$ is the distance of the segment. The steering cost is related to the steering angle, which can be expressed as:

(22) $${\mathrm{cost}}_{s}=\left\{\begin{array}{c} \theta_{j},\;\;\;\theta_{j}\leq \frac{1}{3}\theta_{\max},\\ \frac{k}{2}\theta_{j},\;\;\frac{1}{3}\theta_{\max}<\theta_{j}\leq \frac{2}{3}\theta_{\max},\\ k^{2}\theta_{j},\;\;\frac{2}{3}\theta_{\max}<\theta_{j}\leq \theta_{\max}, \end{array}\right.$$

where *k* is the steering coefficient, and $\theta_{\max}$ is the maximum steering angle.

It is more likely that the movement track will be affected by threat targets such as communication radar, base stations, and buildings. To avoid threat targets, the threat attacking cost ${\mathrm{cost}}_{t}$ should be considered. At first, we calculate the threat degree of each track point as:

(23) $$f_{j}\left(x\right)=\left\{\begin{array}{c} \frac{a^{2}k_{j}^{2}}{d_{xj}^{2}},\;\;\;d_{xj}\leq R_{j},\\ \frac{k_{j}}{d_{xj}^{2}},\;\;\;\;\;R_{j}<d_{xj}\leq bR_{j},\\ 0,\;\;\;\;\;\;\;\;d_{xj}>bR_{j}, \end{array}\right.$$

where $k_{j}$ is the threat level of the threat target, *a* and *b* are the weights of the threat factors, $d_{xj}$ is the distance from the robot to the center of the jth threat target, and $R_{j}$ represents the radius of the threat target. To calculate each threat target in each segment of the total moving track, we evenly divide each segment into 15 parts. The threat attacking cost for mobile robots in each segment is the average value of the robot at the position $x=\frac{2}{15},\frac{4}{15},\frac{6}{15},\frac{8}{15},\frac{10}{15},\frac{12}{15},\frac{14}{15}$. Hence, ${\mathrm{cost}}_{t}$ can be calculated as:

(24) $$cost_{t}=\frac{l_{i}}{7}\sum_{m=1}^{N}\left[\sum_{j=1}^{7}f_{j}\left(\frac{2j}{15}l_{i}\right)\right],$$

where *N* is the number of threat targets.

Moving time cost ${\mathrm{cost}}_{h}$ is connected with the movement time of each segment and can be expressed as:

(25) $${\mathrm{cost}}_{h}=\mu \cdot \Delta T_{i},$$

whereas the movement time factor, $\Delta T_{i}$, is ith segment movement time. The effective cost of the moving track is defined as:

(26) $$f\left(x\right)=\sum_{i=1}^{D+1}\left(\lambda_{1}{\mathrm{cost}}_{t}+\lambda_{2}{\mathrm{cost}}_{e}+\lambda_{3}{\mathrm{cost}}_{h}\right)+\lambda_{4}\sum_{i=1}^{D}{\mathrm{cost}}_{s},$$

where $\lambda_{1},\lambda_{2},\lambda_{3},\lambda_{4}$ represent the weights of ${\mathrm{cost}}_{t},{\mathrm{cost}}_{e},{\mathrm{cost}}_{h},\mathrm{and}{\mathrm{cost}}_{s}$, respectively.

In order to verify the operational efficiency of the algorithm and the effectiveness of the path planning, a simulation on the basis of the Matlab R2020a was conducted to verify the experimental simulation parameters as follows: set each algorithm to run independently 30 times, the pollen population size $N_{p}=300$, and the maximum number of iterations $T_{\max}=1000$. We use PSO, the GA, the FPA, SSO, ABC, and the FPA-CCDE to perform mobile robot path planning. The parameter settings are listed in Table 5, which are also used in [25,26].

Table 6 indicates the time spent in the path planning process of the FPA-CCDE is better than that of the FPA, ABC, PSO, and SSO and slightly worse than that of the GA. The cost of path planning is 65.7% lower than the FPA, 2.8% lower than ABC, 1.7% lower than PSO, 1.7% lower than SSO, and 0.9% lower than the GA. Regarding average variance, the FPA-CCDE is 99.8% lower than the FPA, 3.2% lower than ABC, 63.1% lower than PSO, 80.7% lower than SSO, and 89.8% lower than the GA. It can be seen that in comparison to the other algorithms, the FPA-CCDE can maintain better efficiency in path planning, while consuming the least movement cost, whereas at the same time, the average error of the algorithm is the smallest, and the algorithm is more robust.

**Figure 6:**
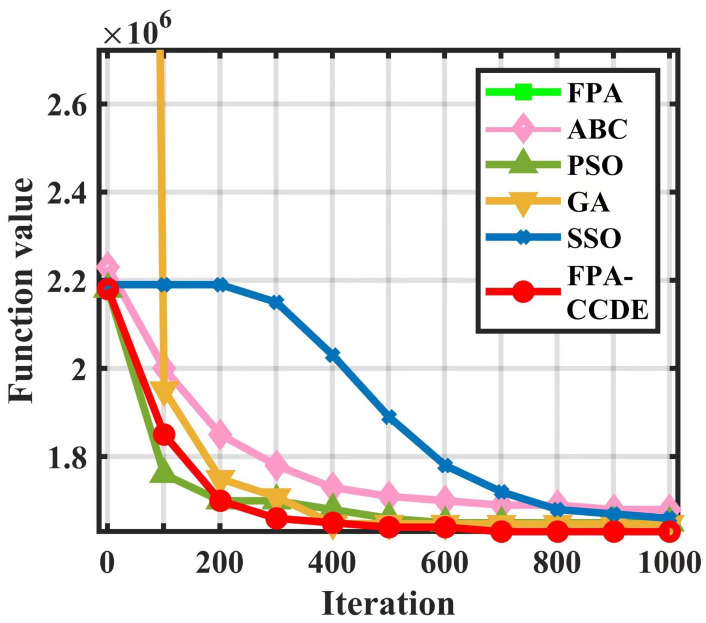
Partial enlargement of the objective function convergence curve.

Figure 6 demonstrates the convergence curve of the objective function of each algorithm as the number of iterations increases. We can see that the ABC algorithm finds the optimal solution on the condition that the number of iterations is 900, and ABC, PSO, and the GA all have the phenomenon of early convergence, which cannot reduce movement expenses. The number of algorithm iterations required for SSO to find the optimal solution is considerably larger than other algorithms. Nonetheless, when the number of iterations of the FPA-CCDE is 700, the value of the objective function does not change. Moreover, the cost of this algorithm is significantly lower than that of the other algorithms.

**Figure 7:**
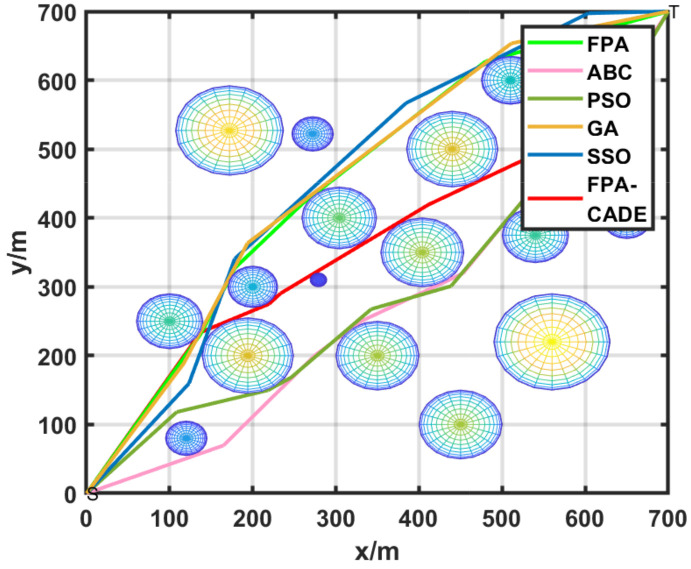
Mobile robot path planning route of two dimensions.

Figure 7 is a top view of the robot path planning. Figure 7 demonstrates that a route from the lower left corner to the upper right corner of the area must be designed in which the buildings are obstacles encountered during the movement, and the colored icons represent the threat targets. Moreover, it can be observed that the SSO and PSO algorithms are highly volatile during the optimization process, and enormous fluctuations occur at the conclusion of the optimization procedure. The FPA-CCDE has better stability in the optimization process, with smooth curves and low volatility. The effect of the FPA-CCDE is superior to the other algorithms.

**Figure 8:**
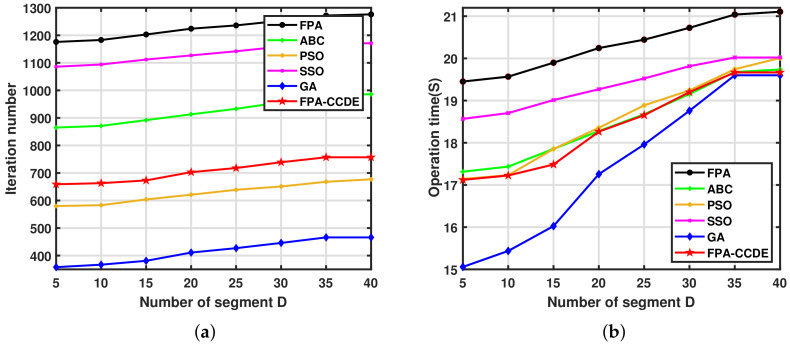
Iterations and operation time under different algorithms with various segments: (**a**) iterations under different segmentation; (**b**) running time under different segmentation.

Figure 8a,b display the number of iterations and running time required for each algorithm to perform path planning under different numbers of segments. Figure 8a demonstrates that as the number of segments D increases, the number of iterations of each algorithm also increases proportionally. When D is set to 35, the number of iterations required by each algorithm to accomplish the path planning tends to be stable. It can be seen from Figure 8b that with the increase in the number of segments, the running time of each algorithm to complete the path planning also increases. When the number of segments D is set to 35, the running time of ABC, SSO, the GA, and the FPA-CCDE to complete the path planning tends to be stable, whereas the execution time of the FPA and PSO increases more rapidly than that of the other algorithms at this time.

**Figure 9:**
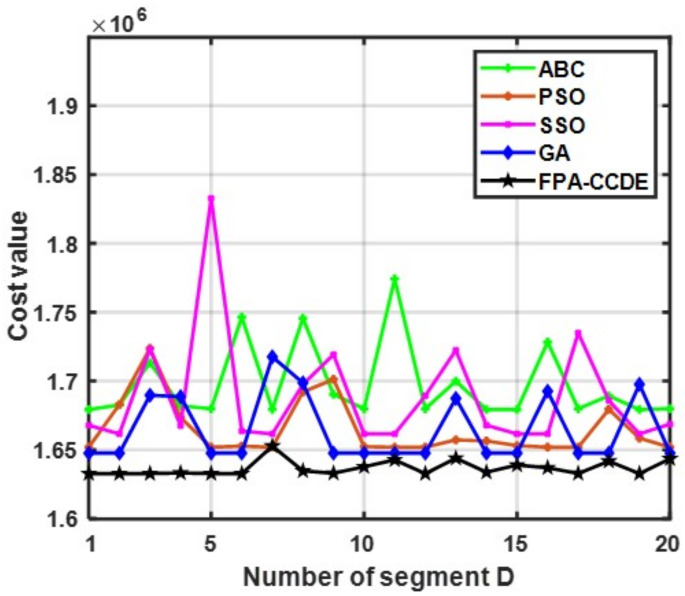
Local amplification of fluctuation.

To verify the stability of these algorithms applied in robot path planning, each algorithm runs independently, and the obtained value of the path planning cost function is demonstrated in Figure 9. The cost value of the FPA-CCDE mostly maintains a stable state, and its value is considerably less than that of the other algorithms.

## 6. Conclusions

In order to solve the optimization problem of high-dimensional variables, this paper designs a flower pollination optimization algorithm based on cosine cross-generation differential evolution. Specifically, individuals are directed to approach the optimal solution by means of differential evolution between generations so that the local searching process of the algorithm is oriented. Setting the cosine inertia weight makes the global search initially strengthen the search ability at a faster rate and enhances the convergence speed of the algorithm. At the same time, the scaling factor and crossover probability are dynamically updated through the parameter adaptive adjustment mechanism, thereby improving the population richness, and the cross-generation roulette method is adopted to reduce the probability of falling into the local optimal solution. Simulation results indicate that the FPA-CCDE displays significant performance advantages in terms of accuracy, average error of algorithm, and stability of the algorithm. In addition, we apply the FPA-CCDE to solve the robot path planning issue. The simulation test demonstrates that our algorithm is capable of low-cost, high-efficiency path planning. In the future, it is expected to be employed in industrial scenarios, such as unmanned submarine path design, automobile cargo distribution route planning, and UAV smart grid fault monitoring.

## Figures and Tables

**Table 1:** Algorithm 1 parameters setting.

Algorithm	Parameters Setting
FPA	P=0.8, λ=1.5
ABC	SN=50
PSO	$c_{1}=2$, $c_{2}=2$, ω=1
SSO	$R_{a}=1$, $P_{c}=0.7$, $P_{m}=0.7$
GA	$P_{c}=0.7$, $P_{m}=0.04$

**Table 4:** F9 with the increase of variables dimension.

	F9 (D = 10)	F9 (D = 30)	F9 (D = 50)	F9 (D = 70)	F9 (D = 100)
FPA-CCDE	**6.2388 × $10^{-28}$** **(1.1777 × $10^{-27}$)**	**1.19848 × $10^{-2}$** **(3.31242 × $10^{-2}$)**	**7.98987 × $10^{-3}$** **(2.76537 × $10^{-2}$)**	**1.19851 × $10^{-2}$** **(3.31241 × $10^{-2}$)**	**1.19853 × $10^{-2}$** **(3.31241 × $10^{-2}$)**
FPA	2.89070 × $10^{-1}$ (5.25541 × $10^{-2}$)	1.18909 × $10^{0}$ (1.76364 × $10^{-1}$)	1.91691 × $10^{0}$ (2.09563 × $10^{-1}$)	2.51704 × $10^{0}$ (2.33520 × $10^{-1}$)	3.12524 × $10^{0}$ (3.38376 × $10^{-1}$)
ABC	2.55878 × $10^{-1}$ (6.50620 × $10^{-2}$)	1.50392 × $10^{0}$ (1.78987 × $10^{-1}$)	2.70389 × $10^{0}$ (1.48553 × $10^{-1}$)	3.64166 × $10^{0}$ (1.22192 × $10^{-1}$)	4.89987 × $10^{0}$ (1.35401 × $10^{-1}$)
PSO	1.59873 × $10^{-1}$ (5.00000 × $10^{-2}$)	4.35873 × $10^{-1}$ (6.37704 × $10^{-2}$)	6.67873 × $10^{-1}$ (7.48331 × $10^{-2}$)	8.39873 × $10^{-1}$ (8.66025 × $10^{-2}$)	1.06387 × $10^{0}$ (7.57188 × $10^{-2}$)
GA	6.45494 × $10^{-1}$ (1.10375 × $10^{-1}$)	1.78149 × $10^{0}$ (1.36208 × $10^{-1}$)	2.60254 × $10^{0}$ (1.28445 × $10^{-1}$)	3.38987 × $10^{0}$ (1.60031 × $10^{-1}$)	4.27658 × $10^{0}$ (1.17499 × $10^{-1}$)
SSO	9.98733 × $10^{-2}$ (2.54849 × $10^{-10}$)	3.03873 × $10^{-1}$ (4.54606 × $10^{-2}$)	5.11873 × $10^{-1}$ (3.31662 × $10^{-2}$)	6.35873 × $10^{-1}$ (4.89898 × $10^{-2}$)	7.59873 × $10^{-1}$ (5.00000 × $10^{-2}$)

**Table 5:** Parameters settings for path planning.

Parameter Name	Parameter Value	Parameter Name	Parameter Value
Movement time factor (μ)	1.5	Maximum angular acceleration ($\varphi_{\max}$:degree)	1.0
Threat factor (*a*)	1.2	Maximum movement distance ($L_{\max}$:metre)	500
Threat factor (*b*)	1.5	Threat attacking cost weight ($\lambda_{1}$)	0.5
Steering coefficient (*k*)	2.5	Energy cost weight ($\lambda_{2}$)	0.3
Maximum turning angle ($\theta_{\max}$)	60	Movement time cost weight ($\lambda_{3}$)	0.5
Maximum linear velocity ($v_{\max}$)	1.0	Steering cost weight ($\lambda_{4}$)	0.3
Maximum angular velocity ($\omega_{\max}$)	0.8	Threat target number (*N*)	18
Maximum linear acceleration ($a_{\max}$)	1.0	Segment number (*D*)	15

**Table 6:** Average objective function of each algorithm.

Algorithm	Convergence Iteration	Time	Running Time	Function Value	Average Error
FPA	1224	20.24543	25.32147	4.76025 × $10^{6}$	1.69787 × $10^{6}$
ABC	913	18.27653	**20.33333**	1.67925 × $10^{6}$	**3.07283 × $10^{3}$**
PSO	621	18.35461	**20.00214**	1.65191 × $10^{6}$	8.06825 × $10^{3}$
SSO	1127	19.26845	21.52612	1.66159 × $10^{6}$	1.54797 × $10^{4}$
GA	411	**17.25684**	20.45647	**1.64760 × $10^{6}$**	2.92722 × $10^{4}$
FPA-CCDE	703	**18.26453**	20.35951	**1.63272 × $10^{6}$**	**2.97392 × $10^{3}$**

## Data Availability

Not applicable.
